# Supplementary figures and images for: Emergent multilevel selection in a simple spatial model of the evolution of altruism
Source: PLoS Comput Biol. 2022 Oct 25;18(10):e1010612. doi: 10.1371/journal.pcbi.1010612 (PMC9595567; doi:10.1371/journal.pcbi.1010612)

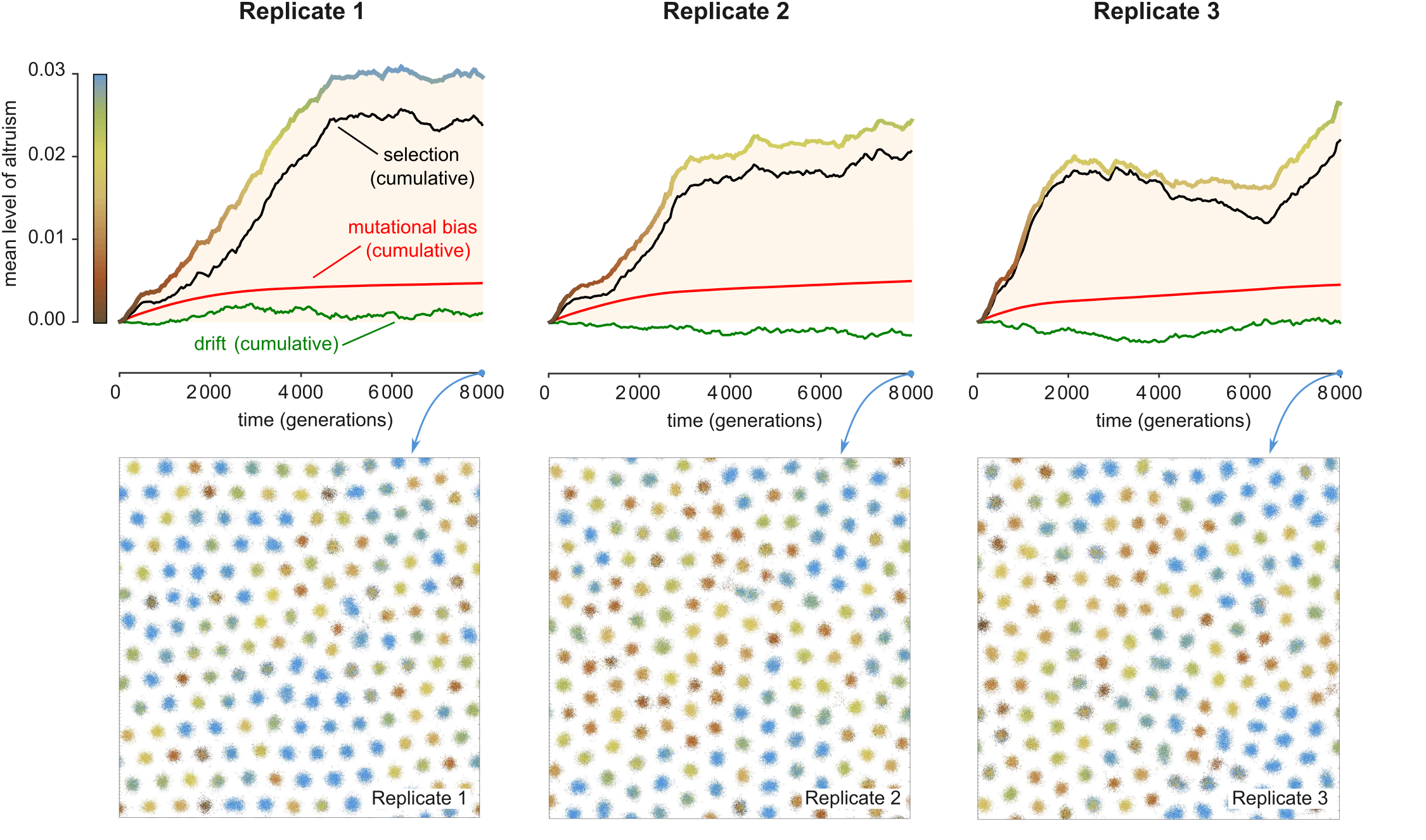

Supplement: S1 Fig — Results are shown based on three simulations that were identical except that the random-number generator was initiated with different random seeds. Fig 2 presents results of Replicate 1. The three figures on top show the mean level of altruism through time (thick colored line). The rise in mean level of altruism can be decomposed into three contributions: natural selection, mutational bias, and random drift, using the method explained in S1 Text Section 3. Plotted are the cumulative contributions of natural selection (black) transmission (red) and genetic drift (green). In all cases, the main contribution is selection, which is consistently positive during the first stretch of the simulations. That said, a mutational bias is revealed as well (red lines). This bias arises because, in this simulation, negative values of ϕ were prohibited (see section Materials and methods) and hence mutations with negative effect are sometimes truncated, especially in individuals with a low trait value. (The smoothness of the red line is a result of the law of large numbers.) The cumulative effect of random drift (green line) is minor in all three replicates. The three figures at the bottom show snapshots of the population at the end of the simulations. In all three cases a hexagonal pattern of colonies has emerged. (TIFF) [file pcbi.1010612.s001.tiff]

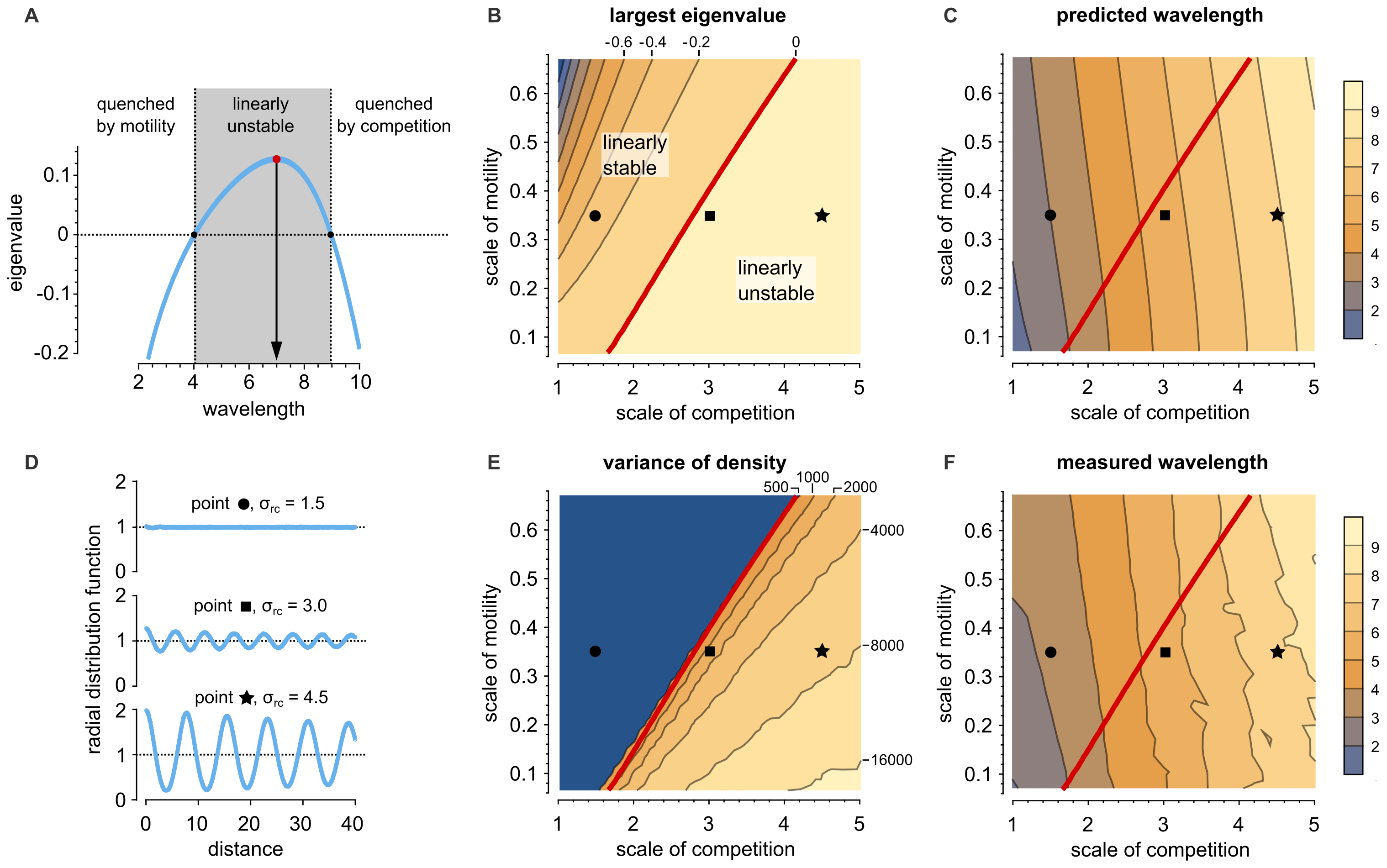

Supplement: S2 Fig — To help understand the conditions for the formation of colonies, a linear stability analysis studies whether, in an initially homogeneous population, small periodic density perturbations tend to grow. (See section Materials and methods for the full derivations.) If they do, this leads to the formation of “colonies”. (A) For given model parameters, each wavelength is associated with an eigenvalue; if the eigenvalue is positive, density waves with this wavelength tend to grow. The figure plots the eigenvalue for a range of wavelengths as calculated for the default parameters of the 1D model (Table 1), additionally assuming all individuals have ϕ = 0.05. Perturbations with small wavelength are quenched by motility; those with a long wavelength by resource competition. In between, a window exists (gray shading) of wavelengths that have a positive eigenvalue. This explains the colony formation in the default parameters. The wavelength with the largest eigenvalue (indicated with the red dot and black arrow) provides a prediction for the wavelength—the distance between neighboring colonies. (B) The largest eigenvalue is plotted as a function of the spatial scales in the system: the scale of motility σm and the scale of competition σrc. (Remember that the scale of altruism σa is 1 by definition of the unit of length.) Otherwise, the assumptions are as in panel A. Colony formation is expected only in the linearly unstable regime, to the right of the red contour line. (C) For the same conditions used in panel B, the predicted wavelength is plotted. As a rule of thumb, it is somewhat smaller than 2σrc. (D) Simulations were performed for the parameters indicated with black symbols in panels B, C, E, and F, assuming that all individuals have an immutable level of altruism ϕ = 0.05. Shown are the resulting radial distribution functions. As expected, the system is nearly homogeneous at σrc = 1.5 (black circle, in the linearly stable regime), weak pair correlations are seen for σrc [file pcbi.1010612.s002.tiff]

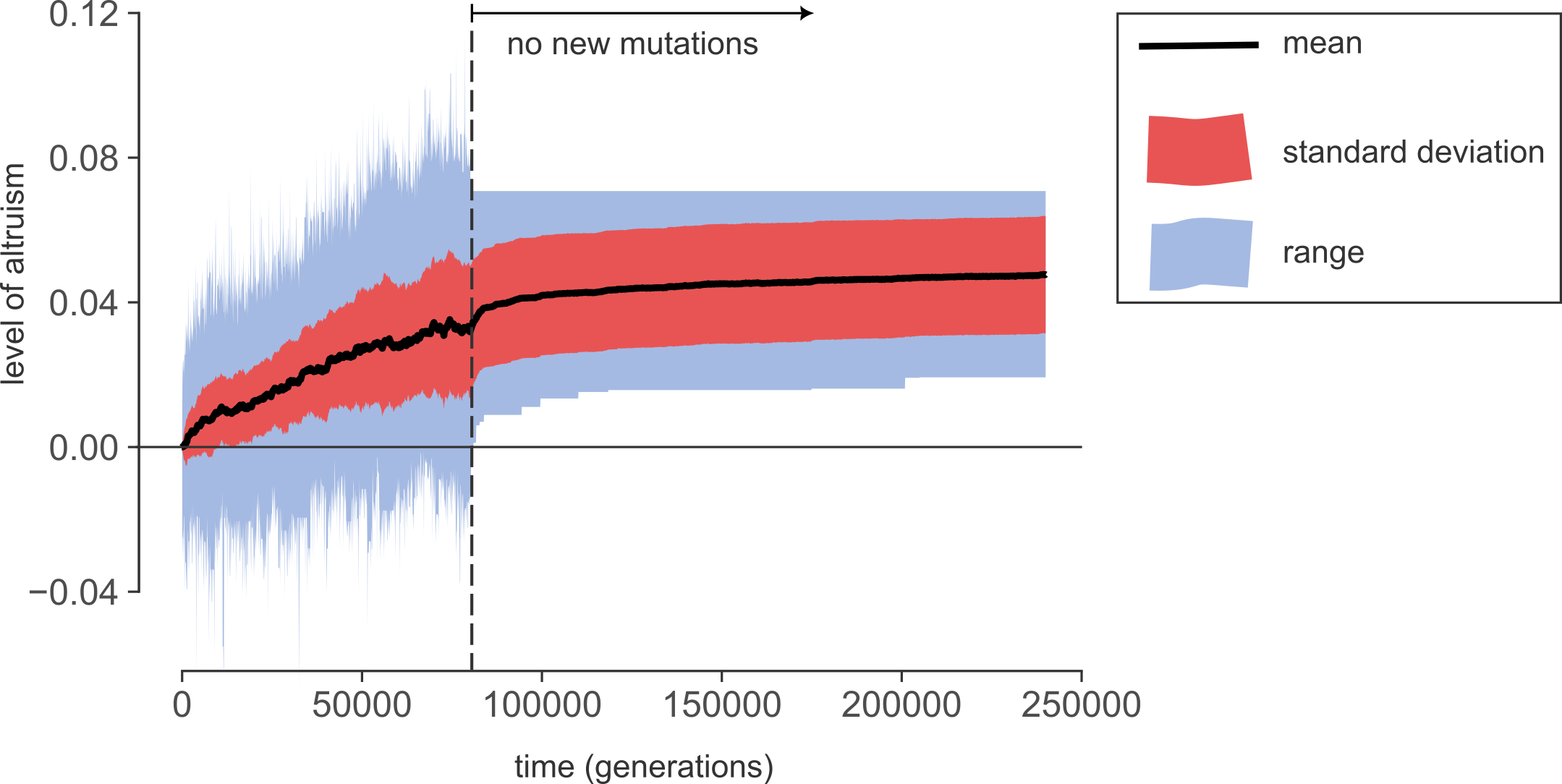

Supplement: S3 Fig — Results are shown for a representative simulation of the 1D model with default parameters. In contrast to other simulation runs, no new mutations are introduced after tnm = 80 000 generations (vertical line). As a result, directly after tnm the range of trait values (blue band) rapidly contracts and defectors (with level of altruism below ∼ 0.01) are eliminated. After this initial decline the distribution of trait values hardly changes: without mutations, the colonies become highly stable and hence between-colony variability is maintained. (TIFF) [file pcbi.1010612.s003.tiff]

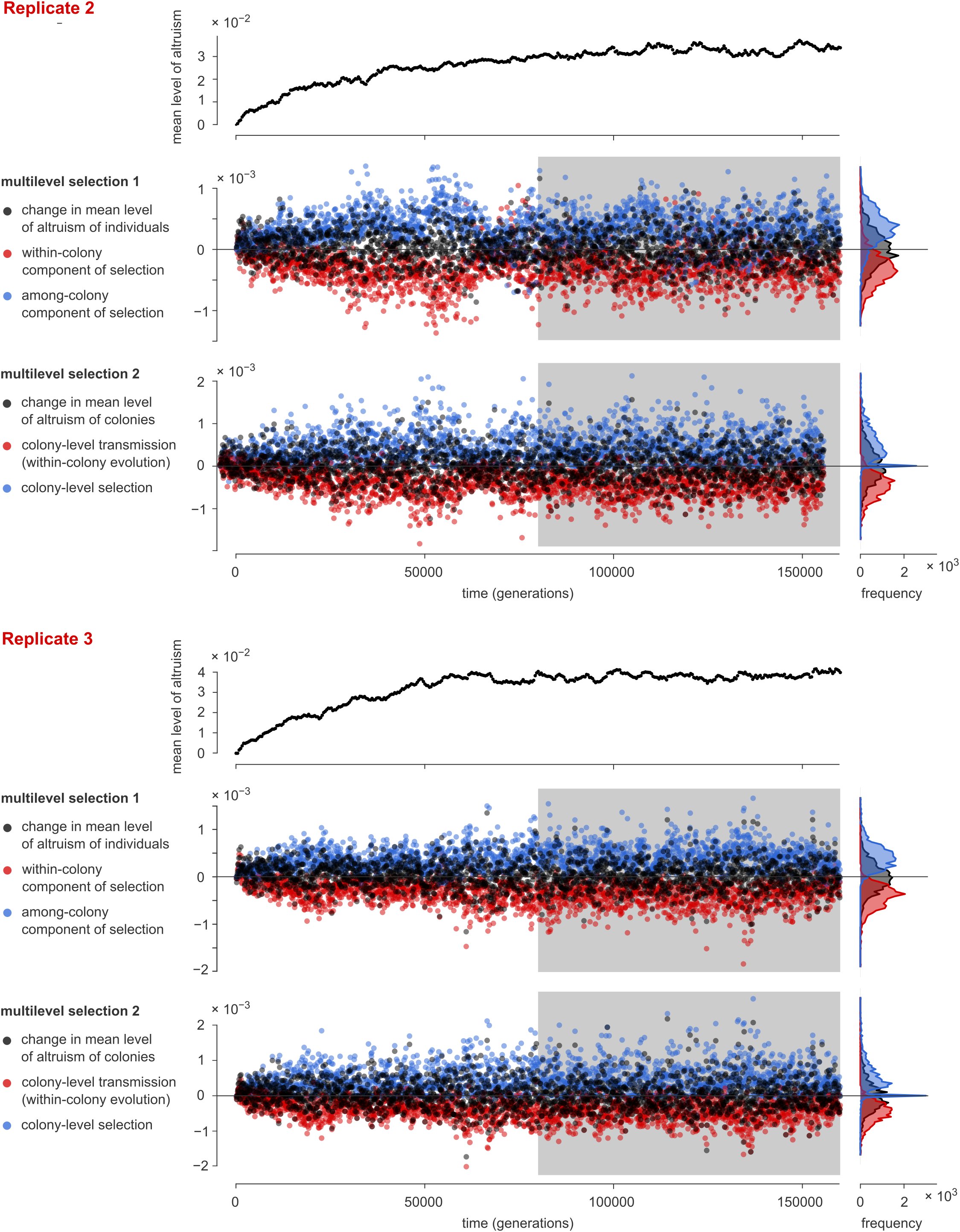

Supplement: S4 Fig — Fig 4 shows results of the quantification of MLS in a single simulation run. To demonstrate the reproducibility of these results, this figure shows the same analysis for two additional replicates. The simulations for all three replicates were identical except that the random-number generator was initiated with a different random seed. All replicates show very similar trends. In particular, the marginal distributions of all quantities are highly consistent. Their statistics are summarized in S1 Table. (TIFF) [file pcbi.1010612.s004.tiff]

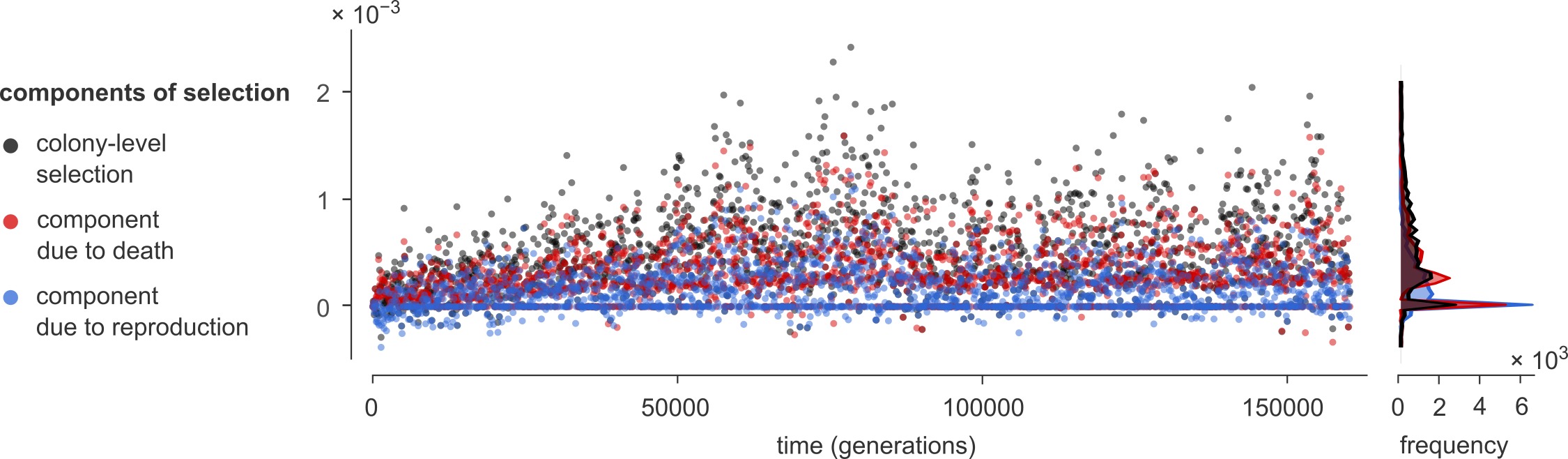

Supplement: S5 Fig — Two mechanisms contribute to the selection of altruistic colonies: they reproduce more frequently and die less frequently. To evaluate the relative importance of these mechanisms we quantify their contribution to the colony-level selection as defined by the MLS 2 analysis; see S1 Text Section 5 and Section 6 for mathematical details. Shown are the results of a single representative simulation with default parameters. As in Fig 4, the analysis was applied to subsequent time intervals of 80 generations (1 000 computational time steps); the figure plots the total colony-level selection (black) as well as the components due to death (red) and reproduction (blue) events. The marginal histograms on the right-hand side are based on the second half of the simulation, after generation 80 000. Note that the components can be precisely zero if no death and/or reproduction events occur in the particular time interval, resulting in considerable overplotting. First, the results demonstrate that both components to selection are much more likely to be positive than negative (Binomial test, p < 10−15 in both cases). The component due to death events was positive in 72.6% and negative in just 1.0% of the time steps, with an average value of (3.2 ± 0.4) × 10−4. The component due to reproduction events was positive in 62.3% and negative in 8.5% of the time steps, with an average value of (1.6 ± 0.2) × 10−4. We conclude that both mechanisms contribute to colony-level selection, but the component due to death is the larger by a factor of two. (TIFF) [file pcbi.1010612.s005.tiff]

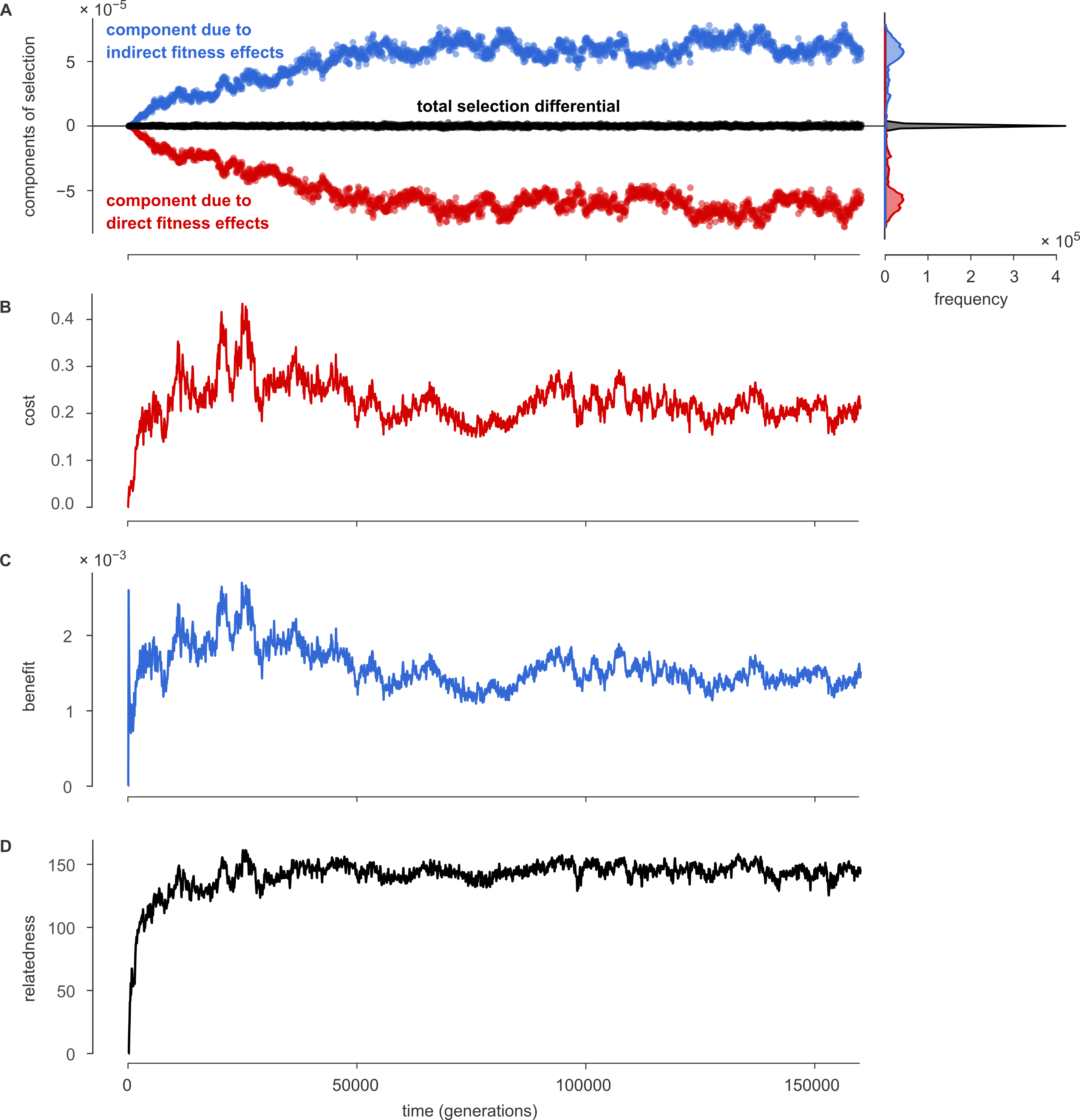

Supplement: S6 Fig — Fig 4 and S4 Fig presented an analysis of the simulations of the 1D model based on multilevel selection theory. Here, it is demonstrated that such simulations can also be analyzed from the perspective of inclusive fitness theory. There are multiple ways to do so; here we essentially adopt a formalism based on a partial regression analysis [68]; see section Materials and methods and S1 Text Section 7 for details. Shown are the results of a single representative simulation with default parameters. In all panels, each data point represents an average of the plotted quantity over the preceding 1000 computational time steps (corresponding to 80 generations). (A) The method splits the selection differential S into two parts. The first part, plotted in red, reflects the selection due to the direct fitness effects: the cost of altruism for the actor. This term is consistently negative, which means that altruists face negative selection due to the direct fitness effects, as expected. The second part, plotted in blue, reflects the selection component due to indirect fitness effects: the benefits of altruism for the recipient. That this term is consistently positive indicates that the benefits must preferentially accrue to more altruistic individuals. Both terms approximately cancel out: the sum of both components S (black) is positive on average but much smaller in absolute value than either term separately. (B) The cost appearing in Hamilton’s rule, measured as a (partial) regression coefficient (S1 Text Section 7), as a function of time. This cost is consistently positive, confirming that altruism is individually costly. (C) The benefit appearing in Hamilton’s rule, demonstrating (as expected) that interacting with altruists yields a benefit. (D) Relatedness among interaction partners is consistently positive. We note that, in the formulation of inclusive fitness theory used here, relatedness can legitimately become much larger than 1 (see S1 Text Section 7). (TIFF) [file pcbi.1010612.s006.tiff]

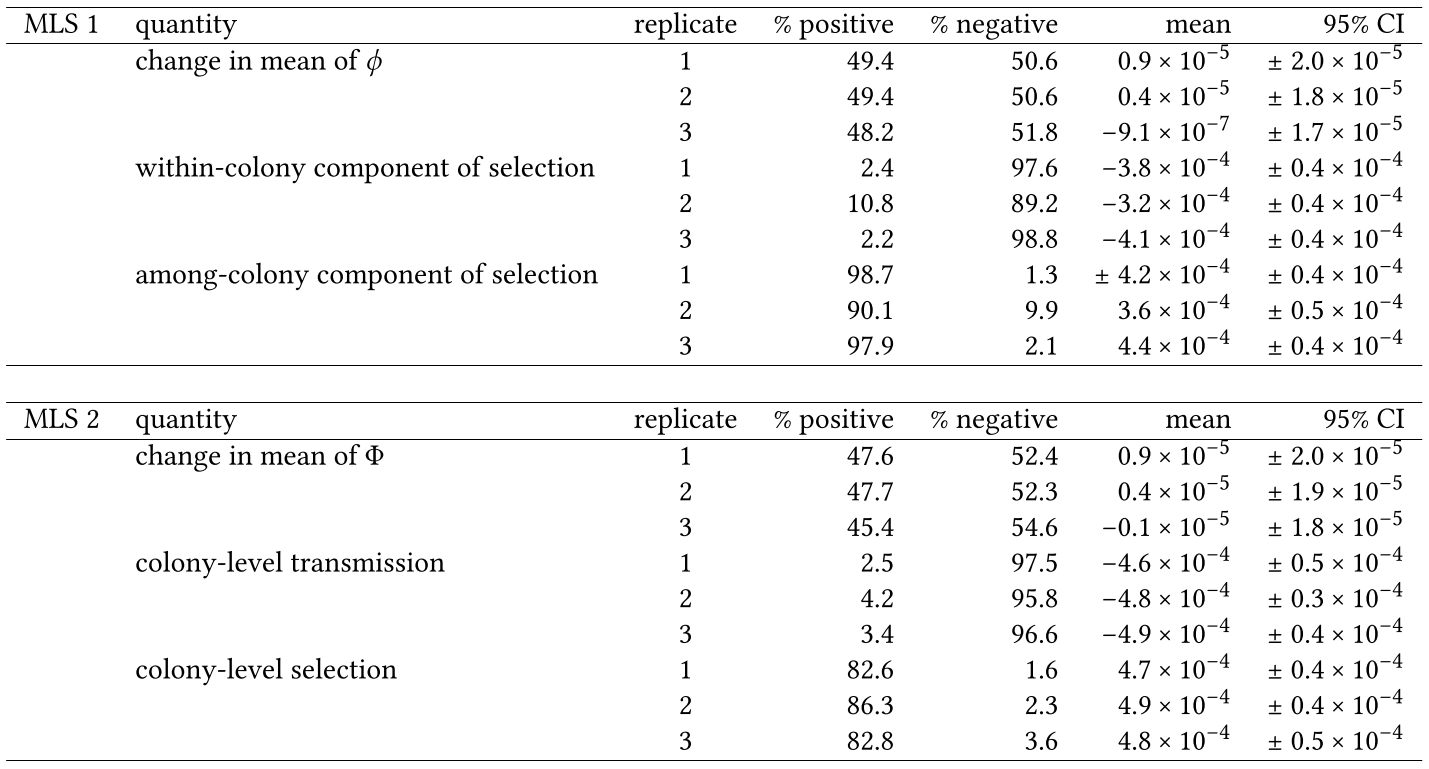

Supplement: S1 Table — Error bars for the means are given as 95% confidence intervals (see section Materials and methods). (TIFF) [file pcbi.1010612.s007.tiff]
