# Supplementary material for: Emergent multilevel selection in a simple spatial model of the evolution of altruism
Source: PLoS Comput Biol. 2022 Oct 25;18(10):e1010612. doi: 10.1371/journal.pcbi.1010612 (PMC9595567; doi:10.1371/journal.pcbi.1010612)
Supplement: S1 Text — In this article, several mathematical results are applied that have been derived long ago. For ease of reference and to facilitate readers who are not intimately familiar with this theory, this text briefly summarizes these results. (PDF) [file pcbi.1010612.s011.pdf]

Evolutionary forces, multilevel selection, and inclusive fitness

S1 Text for:

Emergent multilevel selection in a simple spatial model of the evolution of altruism

Rutger Hermsen[1,2*]

PLOS Computational Biology (2022)

[1] Theoretical Biology Group, Biology Department, Utrecht University, Utrecht, The Netherlands
[2] Centre for Complex Systems Studies, Utrecht University, Utrecht, The Netherlands
[*] r.hermsen@uu.nl

Contents

Contents 1

1 Introduction 1

2 The Price equation 2

3 Measuring selection, random drift, and mutational bias 2

4 Multilevel selection 1 3

5 Multilevel selection 2 4

6 Distinguishing contributions to selection of reproduction and death 5

7 Inclusive fitness theory and Hamilton's rule 6

Bibliography 7

---

1 Introduction

In the main text of the article, several mathematical results are applied that have been derived long ago [1, 2, 3]. For ease of reference and to facilitate readers who are not intimately familiar with this theory, we here briefly summarize these results. Nothing in this document is new (with the possible exception of section 6), although our notation differs somewhat

from other presentations to expose the analogies between the multi*level* selection analysis presented here and the multi*scale* analysis presented elsewhere [4].

2 The Price equation

The Price equation provides a general way to formally describe changes in gene frequencies or mean trait values in evolving populations due to evolutionary forces such as selection and mutation [5, 1, 6].

In its simplest form we consider a population of asexual entities that each possess a numerical trait ϕ. At time t_1, the population size is n, and the population mean of ϕ is $\overline{\phi}$. At a later time $t_2 = t_1 + \Delta t$ the mean of ϕ has changed by an amount $\Delta\overline{\phi}$. Each individual alive at time t_2 has a unique ancestor at time t_1. (If the individual was already born at time t_1, we designate its past self as the ancestor.) Conversely, each individual i alive at time t_1 has W_i offspring at time t_2. (If the individual is itself still alive at time t_2, it is counted as one of the offspring.) W_i is called the absolute fitness of i. The relative fitness w_i of this individual is defined as $w_i = W_i/\overline{W}$, where $\overline{W}$ is the population mean absolute fitness. The trait value ϕ of the offspring of i differs from the value of individual i itself; the average difference among i's offspring is called $\Delta\phi_i$.

With these definitions, the change in the mean value of ϕ over the time interval Δt can be written as:

$$\Delta\overline{\phi} = S + T, \tag{1}$$

with

$$S = \text{Cov}\,(\phi, w) = \overline{\phi w} - \overline{\phi}\,\overline{w}, \tag{2}$$

$$T = \overline{w\Delta\phi}. \tag{3}$$

Equation 1 is called the Price equation. The first term, S, is the population covariance between the trait and relative fitness. It shows that the mean value of ϕ tends to increase if a high value of ϕ is associated with a high fitness. Therefore, S is often considered a measure of the effect of natural selection and called the selection differential. The second term, T, is the average change in trait value between ancestors and their offspring. Therefore T is a measure of transmission bias.

3 Measuring selection, random drift, and mutational bias

Although the Price equation is frequently and fruitfully used in its standard form, it has its limitations. One of these limitations is that it does not acknowledge one of the evolutionary forces central to canonical theory of evolution: random drift.

The absence of random drift from the standard Price equation is a consequence of the definition of fitness used in its formulation. Above, the fitness W_i of individual i was defined as the actual number of offspring it has after the time interval Δt. This is at odds with

the usual parlance, in which fitness refers to an organism's adaptedness to a particular environment. If an organism dies without offspring, this does not necessarily prove that it was poorly adapted to its environment: it might just have been unlucky. The term fitness, then, seems to refer more properly to a propensity or expectation than to an actually realized number of offspring [7, 8]. Deviations from the expectation due to chance are the source of what is usually called random drift.

One way to extend the Price equation is therefore to treat the number of offspring W_i as a random variable and to associate fitness with its expectation value $\mathbb{E}(W_i)$ [3, 6]. In that case we can write the actual number of offspring W_i as $\mathbb{E}(W_i) + \delta W_i$, where δW_i is the deviation from the expectation. If we insert this into the standard Price equation (Eq 1) we arrive at

$$\Delta\overline{\phi} = \underbrace{\mathrm{Cov}\left(\phi, \mathbb{E}(W)/\overline{W}\right)}_{\text{selection}} + \underbrace{\mathrm{Cov}\left(\phi, \delta W/\overline{W}\right)}_{\text{drift}} + \underbrace{\overline{w\Delta\phi}}_{\text{transmission}} . \tag{4}$$

Compared to the standard Price equation, the selection differential S is split into two parts: one part that more properly captures the effects of natural selection, and one term that formalizes random drift.

A complication with the above formulation is that it is not obvious how the probability distribution of W_i, and hence the expectation $\mathbb{E}(W_i)$, should be defined. In particular, it is unclear which variables other than the trait value ϕ should be taken into account — that is, which information the probability distribution should be conditioned on. The more information we incorporate into the expectation, the less uncertainty remains to power random drift. Clearly, this difficult issue is beyond the scope of this work. In the meantime, we take a pragmatic stance: Through convenient choices, the above formalism can be used to examine the contributions of elected sources of randomness, regardless of whether these choices can be justified based on unique "correct" definitions of fitness, selection, and random drift.

4 Multilevel selection 1

Next, we consider a population that is subdivided into N distinct groups. To describe the system from the perspective of MLS 1, we start with the Price equation at the level of the individuals, Eq 1. The idea of the analysis is to split the selection differential S into two parts, S_{within} and S_{among}, where the first accounts for selection taking place *within* groups, and the second for selection *among* groups. We saw that S is defined as a covariance (Eq 2); mathematically, the decomposition is a direct application of the Law of Total Covariance. In the interest of clarity will nevertheless rederive it from scratch.

It will be useful to introduce some notation. Let z be a trait or property of individuals. We will denote the value of z of individual i in group j as z_{ij}, and the size of group j will be written n_j. Then the mean of z within group j is written as $\{z; j\}_{\text{w}}$:

$$\{z; j\}_{\text{w}} \equiv \frac{\sum_{i=1}^{n_j} z_{ij}}{n_j}. \tag{5}$$

The label "w" stands for "within". Whenever this does not give rise to confusion we will omit the group index j and write $\{z\}_{\mathrm{w}}$.

Now, let u be a trait or property of groups. Then we define $\langle u \rangle_{\mathrm{a}}$ as the mean of u among groups, where the groups are weighted according to their group size n_j:

$$\langle u \rangle_{\mathrm{a}} \equiv \frac{\sum_{j=1}^{N} n_j u_j}{n}. \tag{6}$$

The label "a" stands for "among".

From the above definitions, one can verify that

$$\langle \{z\}_{\mathrm{w}} \rangle_{\mathrm{a}} = \overline{z}. \tag{7}$$

That is to say, if we know the mean value of z within each group, $\{z\}_{\mathrm{w}}$, we can recover the population mean $\overline{z}$ by averaging the over all groups, provided we give larger groups a larger weight.

With the above notation and Eq 7 in place, the decomposition of S is obtained quite directly:

$$\begin{aligned} S = \mathrm{Cov}\,(\phi, w) &= \overline{\phi w} - \overline{\phi}\,\overline{w} \\ &= \left\langle \{\phi w\}_{\mathrm{w}} \right\rangle_{\mathrm{a}} - \left\langle \{\phi\}_{\mathrm{w}} \right\rangle_{\mathrm{a}} \langle \{w\}_{\mathrm{w}} \rangle_{\mathrm{a}} \\ &= \left\langle \{\phi w\}_{\mathrm{w}} \right\rangle_{\mathrm{a}} - \left\langle \{\phi\}_{\mathrm{w}} \{w\}_{\mathrm{w}} \right\rangle_{\mathrm{a}} + \left\langle \{\phi\}_{\mathrm{w}} \{w\}_{\mathrm{w}} \right\rangle_{\mathrm{a}} - \left\langle \{\phi\}_{\mathrm{w}} \right\rangle_{\mathrm{a}} \langle \{w\}_{\mathrm{w}} \rangle_{\mathrm{a}} \\ &= \left\langle \{\phi w\}_{\mathrm{w}} - \{\phi\}_{\mathrm{w}} \{w\}_{\mathrm{w}} \right\rangle_{\mathrm{a}} + \left\langle \{\phi\}_{\mathrm{w}} \{w\}_{\mathrm{w}} \right\rangle_{\mathrm{a}} - \left\langle \{\phi\}_{\mathrm{w}} \right\rangle_{\mathrm{a}} \langle \{w\}_{\mathrm{w}} \rangle_{\mathrm{a}} \\ &= \langle \mathrm{Cov}_{\mathrm{w}}\,(\phi, w; j) \rangle_{\mathrm{a}} + \mathrm{Cov}_{\mathrm{a}} \left(\{\phi\}_{\mathrm{w}}, \{w\}_{\mathrm{w}} \right) \\ &\equiv S_{\mathrm{within}} + S_{\mathrm{among}}. \end{aligned} \tag{8}$$

Here we introduced $\mathrm{Cov}_{\mathrm{w}}\,(y, z; j) \equiv \{yz; j\}_{\mathrm{w}} - \{y; j\}_{\mathrm{w}} \{z; j\}_{\mathrm{w}}$ as the covariance between individual properties y and z as measured within group j, and $\mathrm{Cov}_{\mathrm{a}}\,(u, v) = \langle uv \rangle_{\mathrm{a}} - \langle u \rangle_{\mathrm{a}} \langle v \rangle_{\mathrm{a}}$ as the covariance of group properties u and v among groups, where groups are weighted by their group size.

Eq 8 shows that S_{within} quantifies to what extent within groups the trait value ϕ is associated with fitness. It can hence be interpreted as the effect of selection taking place within groups. On the other hand, S_{among} measures whether groups with a high mean of ϕ tend to have a high mean fitness. It can hence be interpreted as the selection component that results from selection among groups.

5 Multilevel selection 2

We note that the calculations for MLS 1 can be executed for subdivided populations regardless of whether the groups themselves can in any meaningful way be said to reproduce or die. An alternative formalism, called MLS 2, does explicitly require reproduction at the level of groups.

The idea of MLS 2 is that, if the groups themselves reproduce, the Price equation can be applied at the level of groups. Now the relevant population is the population of groups, and

the Price equation can describe the evolution of any trait Φ that is a property of groups:

$$\Delta\overline{\Phi} = \underbrace{\operatorname{Cov}(\Phi, \omega)}_{\text{group-level selection}} + \underbrace{\overline{\omega\Delta\Phi}}_{\text{group-level transmission}} . \quad (9)$$

Importantly, the relative fitness ω_j in this Price equation now represents the fitness of group j, that is, the (relative) number of groups at time t_2 that are its offspring (including the group itself, if it survives until t_2).

If we are interested in the evolution of a particular trait at the individual level ϕ — such as the level of altruism — we are free to choose Φ to be the group mean of ϕ; that is, $\Phi_j = \{\phi; j\}_{\mathrm{w}}$. The first term in Eq 9 then measures the effect of selection at the group level on the mean trait value of groups. The second term quantifies the effect of bias in the changes in Φ between ancestral groups and their offspring; this reflects the internal evolution of groups.

6 Distinguishing contributions to selection of reproduction and death

We saw that the Price equations is always applied to a particular time interval $(t_1, t_2]$. The fitness of an organism or group at time t_1 was defined as the number of offspring it has at time t_2. These fitnesses are therefore determined by two types of events: reproduction and death. It seems clear that natural selection could result from the association of a trait with either of these types of events; hence, we should be able to separate the effects of death and reproduction on selection.

To do so, we note that absolute fitness W_i of individual i can be written as follows:

$$W_i = 1 + n_{\mathrm{r},i} - n_{\mathrm{d},i}, \quad (10)$$

where $n_{\mathrm{r},i}$ and $n_{\mathrm{d},i}$ respectively represent the number of reproduction and deaths events occurring withing the lineage starting with individual i during the time interval $(t_1, t_2]$. (Here, events involving i itself should be counted as well.) Inserting this into the expression for the selection differential $S = \operatorname{Cov}\left(\phi, W/\overline{W}\right)$ directly gives:

$$S = \underbrace{\operatorname{Cov}(\phi, n_{\mathrm{r}})/\overline{W}}_{\text{selection component due to reproduction}} \underbrace{- \operatorname{Cov}(\phi, n_{\mathrm{d}})/\overline{W}}_{\text{selection component due to death}} . \quad (11)$$

These two terms measure whether the trait is associated with reproduction or death, respectively.

Eq 11 dissects selection at the individual level, but if we substitute individual-level quantities by their group-level analogues, the same analysis can be applied to the group-level selection term of MLS 2 to assess the effects of death and reproduction of *groups* on the selection of *groups*.

7 Inclusive fitness theory and Hamilton's rule

Within inclusive fitness theory, a variety of approaches and formalisms have been developed [9, 10]. Here we will merely derive the approach used for the analysis of S6 Fig, which is based on Ref. [11] by Queller.

The idea is to write relative (neighbor-modulated) fitness in the following linear-regression form:

$$w_i = w_0 - \beta_c \phi_i + \beta_b \phi_i' + \epsilon_i. \tag{12}$$

Here, ϕ_i is the trait value of individual i, and ϕ_i' is the sum of the trait values of all individuals that i interacts with. The intercept W_0 and the partial regression coefficients β_i are obtained by minimizing the sum of the squared residuals ϵ_i.

It is important to note that we do *not* assume that that w truly is a linear function of ϕ and ϕ'. Whether or not w is linear, the linear fit of Eq 12 always exists (provided ϕ and ϕ' vary within the population and are not collinear); non-linearities are absorbed by the residuals. Also, we do *not* assume that the residuals are independent and normally distributed. Such assumptions are often made in the context of statistical hypothesis tests based on linear models because they conveniently ensure that the desired p-values can be calculated from t or F distributions. Here, it is not our intention to perform a statistical hypothesis test and hence there is no reason to make these assumptions.

With this in mind, parameter β_c expresses whether, as a trend, fitness tends to increase or decrease with ϕ if ϕ' is kept constant. It can therefore be interpreted as a measure of the cost of the trait. (The minus sign in Eq 12 was chosen to ensure that a positive β_c indicates that the trait comes at a cost.) Similarly, β_b measures to what extend fitness tends to increase with ϕ' (as ϕ is held constant) and hence summarizes the benefit of the trait to social partners.

Next, we insert Eq 12 in Eq 2, the expression for the selection differential S. The term $\mathrm{Cov}(\phi, \epsilon)$ vanishes because, as a general property of least-square linear fits, the covariance between the residuals and each of the regression variables is zero. The result is:

$$S = \underbrace{-\beta_c \mathrm{Var}(\phi)}_{\text{direct fitness effects}} + \underbrace{\beta_b \mathrm{Cov}(\phi, \phi')}_{\text{indirect fitness effects}}. \tag{13}$$

The first term in Eq 13 represents the component of selection resulting from direct fitness effects, *i.e.*, costs to the actor. The second term represents selection resulting from indirect fitness effects.

A trait is under positive selection if $S > 0$. From Eq 13 this can be rewritten as

$$\underbrace{\beta_c}_{\text{cost}} < \underbrace{\beta_b}_{\text{benefit}} \underbrace{\left(\frac{\mathrm{Cov}(\phi, \phi')}{\mathrm{Var}(\phi)} \right)}_{\text{relatedness}}. \tag{14}$$

The factor labeled "relatedness" takes the form of a regression coefficient between the trait value ϕ of the actor and the total trait value of its interaction partners ϕ' . Hence, this is a version of Hamilton's rule. It states that a costly social trait can nevertheless be selected provided the benefits are large enough and accrue disproportionately to individuals with a high trait value.

If individuals interact with multiple interaction partners, ϕ' can on average be considerably larger than ϕ, so that the relatedness factor in Eq 14 may become (much) larger than one. In addition, it can in theory assume negative values. This is in contrast to some popular alternative versions of Hamilton's rule, in which relatedness is confined to the interval $[0, 1]$.

Bibliography

[1] Price GR. Extension of Covariance Selection Mathematics. Annals of Human Genetics. 1972;35(4):485–490.

[2] Damuth J, Heisler IL. Alternative Formulations of Multilevel Selection. Biology and Philosophy. 1988;3(4):407–430. doi:10.1007/BF00647962.

[3] Okasha S. Evolution and the Levels of Selection. Oxford University Press; 2006.

[4] Doekes HM, Hermsen R. Multiscale Selection in Spatially Structured Populations; 2021.

[5] Price GR. Selection and Covariance. Nature. 1970;227(5257):520–521. doi:10.1038/227520a0.

[6] Rice SH. Evolutionary Theory: Mathematical and Conceptual Foundations. Sinauer Associates; 2004.

[7] Brandon RN. Adaptation and Evolutionary Theory. Studies in History and Philosophy of Science Part A. 1978;9(3):181–206. doi:10.1016/0039-3681(78)90005-5.

[8] Mills SK, Beatty JH. The Propensity Interpretation of Fitness. Philosophy of Science. 1979;46(2):263–286. doi:10.1086/288865.

[9] Frank SA. Foundations of Social Evolution. Princeton: Princeton University Press; 1998.

[10] Marshall JAR. Social Evolution and Inclusive Fitness Theory : An Introduction. Princeton: Princeton University Press; 2015.

[11] Queller DC. A General Model for Kin Selection. Evolution. 1992;46(2):376–380. doi:10.2307/2409858.
